# CHIR99021-Treated Osteocytes with Wnt Activation in 3D-Printed Module Form an Osteogenic Microenvironment for Enhanced Osteogenesis and Vasculogenesis

**DOI:** 10.3390/ijms24066008

**Published:** 2023-03-22

**Authors:** Yisheng Luo, Yangxi Liu, Bo Wang, Xiaolin Tu

**Affiliations:** Laboratory of Skeletal Development and Regeneration, Institute of Life Sciences, Chongqing Medical University, Chongqing 400016, China; a554551974@163.com (Y.L.); 2020111818@stu.cqmu.edu.cn (Y.L.); 2019111169@stu.cqmu.edu.cn (B.W.)

**Keywords:** osteocyte, osteogenic microenvironment, COOME, bone marrow stromal cells, CHIR99021

## Abstract

Finding a bone implant that has high bioactivity that can safely drive stem cell differentiation and simulate a real in vivo microenvironment is a challenge for bone tissue engineering. Osteocytes significantly regulate bone cell fate, and Wnt-activated osteocytes can reversely regulate bone formation by regulating bone anabolism, which may improve the biological activity of bone implants. To achieve a safe application, we used the Wnt agonist CHIR99021 (C91) to treat MLO-Y4 for 24 h, in a co-culture with ST2 for 3 days after withdrawal. We found that the expression of Runx2 and Osx increased, promoted osteogenic differentiation, and inhibited adipogenic differentiation in the ST2 cells, and these effects were eliminated by the triptonide. Therefore, we hypothesized that C91-treated osteocytes form an osteogenic microenvironment (COOME). Subsequently, we constructed a bio-instructive 3D printing system to verify the function of COOME in 3D modules that mimic the in vivo environment. Within PCI3D, COOME increased the survival and proliferation rates to as high as 92% after 7 days and promoted ST2 cell differentiation and mineralization. Simultaneously, we found that the COOME-conditioned medium also had the same effects. Therefore, COOME promotes ST2 cell osteogenic differentiation both directly and indirectly. It also promotes HUVEC migration and tube formation, which can be explained by the high expression of *Vegf*. Altogether, these results indicate that COOME, combined with our independently developed 3D printing system, can overcome the poor cell survival and bioactivity of orthopedic implants and provide a new method for clinical bone defect repair.

## 1. Introduction

Globally, many reconstructive surgeries are required each year to address bone defects caused by accidental fractures, osteoporosis, cancer, and genetic disorders such as chondromalacia [1]. Although bone tissue has strong reparative and regenerative abilities, borderline bone defects caused by trauma, tumors, or infections do not heal on their own and patients with these issues require bone grafting [2]. This has severely increased the burden on socioeconomic and healthcare systems; therefore, bone defect repair remains an imminent challenge for orthopedic surgeons. The current mismatch in anatomical characteristics and lack of bioactivity in autogenous, allogeneic, and artificial bone grafts result in instability, loosening, and the need for renovation [3,4]. These solutions are inadequate for clinical needs, but the rapid development in bone tissue engineering (BTE) offers hope.

Three-dimensional printing technology allows the fabrication of scaffolds with a predetermined external structure, internal pore size, porosity, and pore connectivity [5]; furthermore, the precise design of the scaffold geometry meets the requirements for the optimization of implant materials for bone defect repair [6,7,8]. For a better functional osteogenesis, growth factors, such as VEGF, BMP, and other biological components, have been combined with materials to construct bone implants [9,10]. However, significant side effects and severe complications exist; for example, recombinant human bone morphogenetic protein-2 (rhBMP-2) and rhBMP-7 are used clinically to promote bone healing but are applied at very high doses, which can lead to heterotopic bone formation and cyst-like bone formation, inflammation, and swelling. These effects also increase economic burden [11,12]. In 2016, a new tissue organ printer (ITOP) available for material building and cell-integrated printing became available. The 3D functional modules constructed by this printer formed vascularized bone after implantation into parietal bone defect sites in mice [13], paving the way for improved BTE in the future. However, a key drawback is the lack of osteoinductive activity, making it difficult to accelerate the osteogenic process.

In recent years, the biological functions of the cellular microenvironment have been recognized to play a crucial role in maintaining the homeostasis, repair, and regeneration of skeletal tissues [14]. This crucial role inspired us to combine 3D printing with the cellular microenvironment to create functional modules with biological activity. In addition to regulating bone homeostasis by integrating mechanical load and hormonal signals, osteocytes also control bone anabolism by influencing osteoblasts and osteoclasts through paracrine signaling, suggesting that terminally differentiated osteocytes can inversely regulate bone formation [15].

Numerous studies have suggested that the classical Wnt signaling pathway is positively involved in bone formation [16,17,18]. Wnt ligands bind to the frizzled and LRP5/6 receptors and reduce β-catenin degradation in the cytoplasm. Then, stable β-catenin accumulates in the cell nucleus and binds to the TCF/LEF transcription factors, which in turn activates target gene expression [19,20]. Previous studies have found that in vivo activation of Wnt signaling in osteocytes regulates bone formation and bone resorption, which enables bone metabolism [15] and promotes vascular and neural differentiation [21]. To replace gene editing for clinical applications, our team focuses on finding small molecule drugs that can stably activate signaling pathways and on developing safe and effective bone regeneration and repair solutions. CHIR99021 (C91) is a highly specific, safe, and effective GSK-3 inhibitor that is widely used as a Wnt activator [22,23,24]. In this study, we found that C91 at 2.5 µM and 5 µM activated Wnt signaling in osteocytes without affecting cell proliferation activity and that C91-treated osteocytes contributed to bone differentiation, angiogenesis, and inhibition of adipogenesis. According to these results, we hypothesized that C91-treated osteocytes may form a safe and effective osteogenic microenvironment (COOME), which was further verified in COOME 3D modules.

Our team constructed 3D modules with PCL and cell-integrated 3D printing systems (PCI3D) [25]. These allowed us to address the bottleneck issue of cell survival and growth on hard materials because the PCI3D module steadily supports cell growth in scaffolds by providing a suitable pore size, which allows the easy transport and excretion of nutrients indispensable for cell growth. Additionally, the main innovation of this study is the use of osteocytes that activate Wnt signaling to promote osteogenic differentiation and mineralization of bone marrow stromal cells, which is safer than direct drug administration. Based on C91-treated osteocytes, we constructed a COOME 3D functional module. Three-dimensional culture mimicking in vivo environments revealed that COOME was beneficial for cell survival and proliferation on PCL, as proven by the 92% survival rate after 7 days of culture. It also significantly promoted cell proliferation and supported osteoblast differentiation and mineralization. This strategy solved the problem of poor cell survival and proliferation on hard materials that have existed in bone tissue engineering (BTE). In addition, COOME promoted HUVEC cell migration and angiogenesis and inhibited adipogenesis. This study suggests that the design of hard materials and cell-integrated 3D printing combined with an osteogenic microenvironment is a candidate strategy for BTE for the regenerative repair of bone defects.

## 2. Results

### 2.1. C91 Activates the Wnt/β-Catenin Signaling Pathway in the Osteoblast Cell Line MLO-Y4

**Figure 1:**
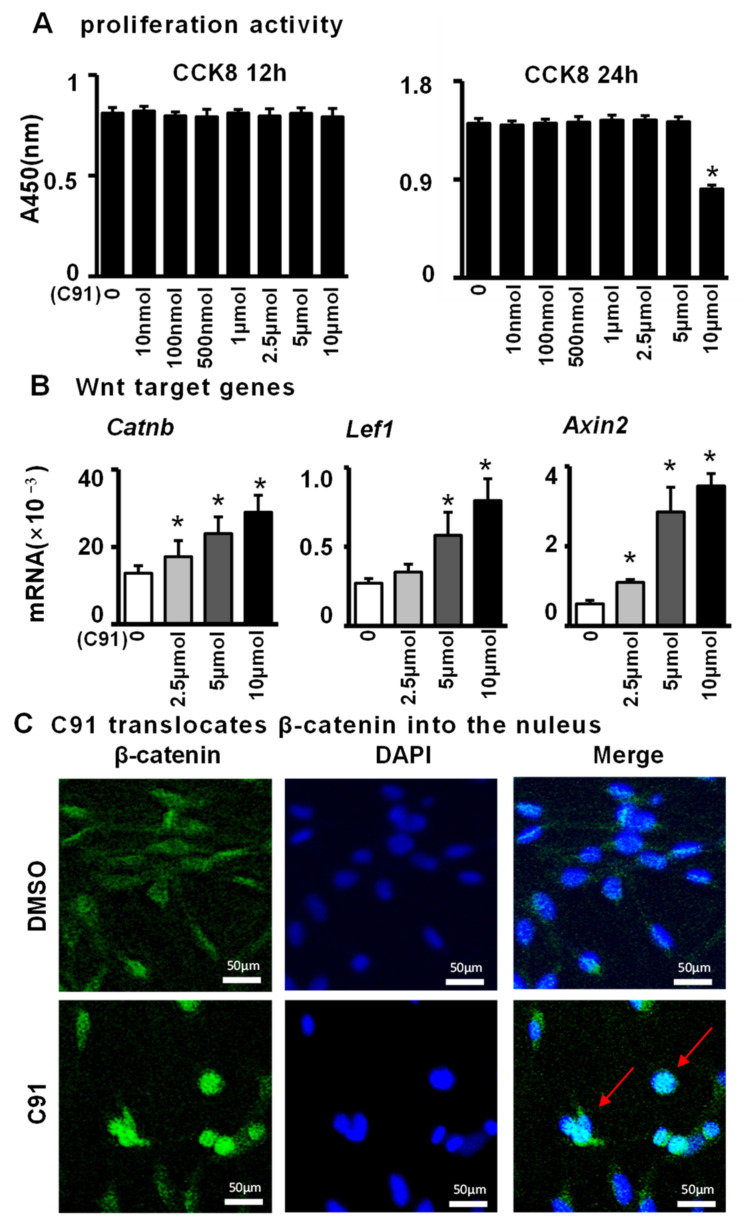
The effects of C91 on the Wnt/β-catenin signaling pathway in MLO-Y4 cells. (**A**) Using the CCK8 assay to test the effect of C91 on the proliferation of MLO-Y4 cells at 12 h or 24 h. Results are expressed as relative OD450 values. Amounts of 0–5 µM of C91 for 12 h did not significantly influence the MLO-Y4 cell proliferation activity. (**B**) qPCR detection of Wnt target gene expression. (**C**) MLO-Y4 cells were treated with 5 µM of C91 for 24 h, and the samples were collected for immunofluorescence staining analysis of β-catenin. The red arrows indicate the protein of β-catenin into the nucleus. Scale bar = 50 µm. Results are expressed as mean ± SD (n = three per group). * indicates *p* < 0.05 vs. the control group by one-way ANOVA.

C91 is well known as a Wnt agonist; however, we needed to determine the optimal concentration for activating Wnt signaling in the osteocyte cell line MLO-Y4. The CCK-8 assay revealed (Figure 1A) the effects of different concentrations (0, 10 nM, 100 nM, 500 nM, 1 µM, 2.5 µM, 5 µM, 10 µM) of C91 treatment on MLO-Y4 cells for 12 h and 24 h. The results showed that 0–5 µM of C91 did not cause changes in cell proliferation activity. However, when MLO-Y4 cells were treated with 10 µM C91 for 24 h, the proliferation activity of MLO-Y4 cells was significantly reduced (*p* < 0.05). We then treated MLO-Y4 cells with C91 at concentrations of 0, 2.5, 5, and 10 µM for 12 h and 24 h for qPCR. The results confirmed that C91 upregulated the Wnt target genes *Catnb*, *Lef1*, and *Axin2* in a time- and dose-dependent manner (Figure 1B). To further determine whether C91 activates Wnt signaling through the classical pathway, we tested the level of β-catenin in the nucleus by immunofluorescence with an anti-β-catenin antibody. As expected, the fluorescence density and intensity in the nucleus were significantly higher after C91 treatment for 24 h than in the control group (Figure 1C). Therefore, in all subsequent experiments, either 2.5 µM or 5 µM of the C91 drug concentration was used.

### 2.2. C91-Induced Osteocytes Promote Osteoblast Differentiation and Mineralization of ST2 Cells via Activation of Wnt Signaling

**Figure 2:**
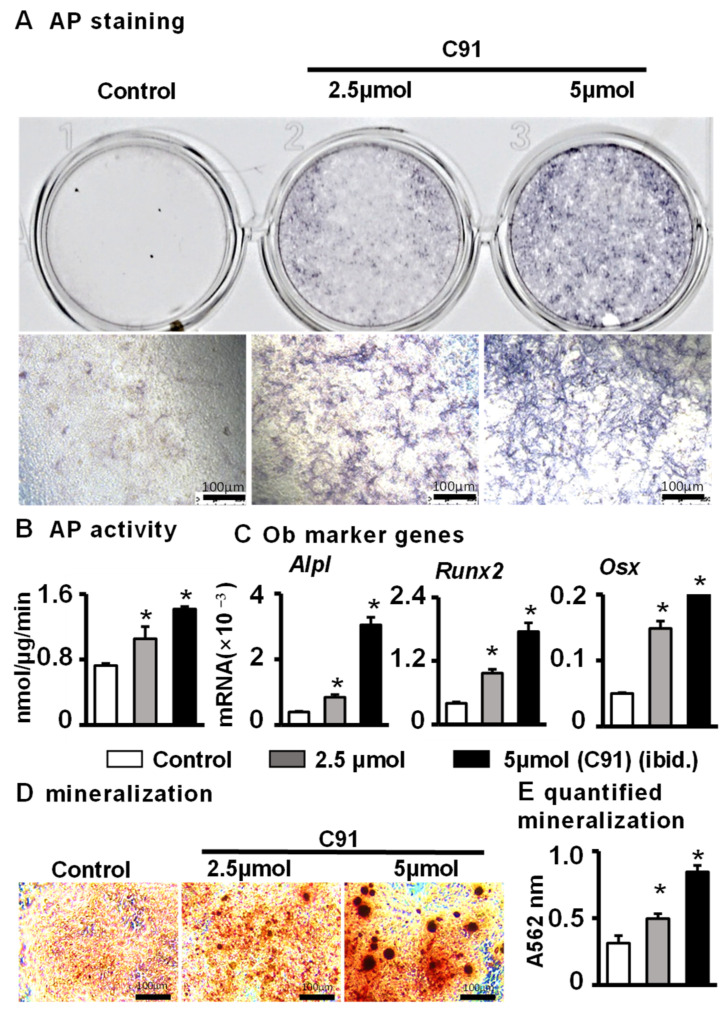
The effects of MLO-Y4 treated by C91 on osteogenic differentiation and mineralization in ST2 cells. After 24 h of treatment of 2.5 µM or 5 µM C91, MLO-Y4 was co-cultured with ST2 cells in the new medium for 3 days, followed by (**A**) AP staining analysis and (**B**) AP biochemical quantification analysis. (**C**) qPCR was used for the detection of the mRNA expression level of osteogenesis marker genes. (**D**) ST2 cells co-cultured with COOME were cultured in the growth medium for 3 days and in the osteogenic medium for another 14 days. Alizarin red S staining and quantification assay (**E**) were used for detecting the mineralized bone nodules. Results are expressed as mean ± SD (n = three per group). Scale bar = 100 µm. * indicates *p* < 0.05 vs. the control group by one-way ANOVA.

Our previous studies confirmed that either 2.5 or 5 µM of C91 can lead to a significant activation of Wnt signaling in osteocytes. To detect the effect of treated osteocytes on the osteogenic differentiation of ST2 cells, we treated the osteocyte cell line MLO-Y4 with different concentrations of C91 (0 µM, 2.5 µM, 5 µM) for 24 h, then withdrew the drug and co-cultured these cells with ST2 cells for 3 days to observe the osteogenic differentiation of ST2 cells. AP staining results and quantitative analysis showed that AP activity in the C91-treated group was much higher than that in the control group (Figure 2A,B). The qPCR results showed that osteocyte-activated Wnt signaling significantly promoted the mRNA expression of the osteoblast marker genes *Alpl*, *Runx2*, and *Osx* (Figure 2C). In addition, the results of mineralization induction experiments showed that the C91-treated group formed larger and denser mineral nodules than the control group (Figure 2D,E). These data indicate that C91-treated osteocytes formed COOME that promotes osteoblast differentiation.

### 2.3. COOME Inhibits Adipocyte Formation

**Figure 3:**
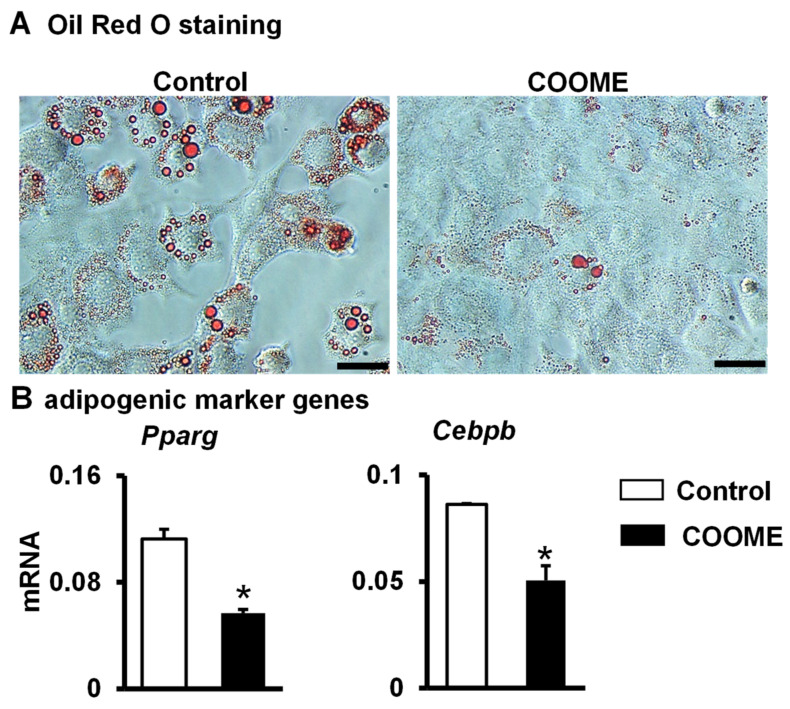
The effect of COOME on ST2 cells adipogenic differentiation. ST2 cells co-cultured with COOME in an adipogenic culture medium until lipid droplets were formed. (**A**) Oil red O staining. Scale bar = 20 µm. (**B**) qPCR detection of mRNA expression of adipogenic differentiation marker genes. Results are presented as mean ± SD (n = three per group). * indicates *p* < 0.05, vs. the control group.

MSCs have multidirectional differentiation potential, including the ability to differentiate into osteoblasts or adipocytes [26,27]. Studies have reported that there is an antagonistic relationship between adipogenesis and osteogenesis [28,29]. Our results showed that COOME could promote osteogenic differentiation; therefore, we further verified the effect of COOME on adipocyte differentiation. We used an adipogenic induction medium to induce ST2 cell differentiation. Oil red O staining showed that the formation of lipid droplets in the COOME group was less than that in the control group (Figure 3A), and qPCR results showed that the expression of peroxisome proliferator-activated receptor γ (*Pparg*) and *Cebpb* (Figure 3B), the main transcriptional regulators of adipocyte differentiation, were down-regulated. It is hypothesized that MLO-Y4 cells with activated Wnt signaling may inhibit the adipogenic differentiation of ST2 cells by downregulating the expression of *Pparg* and *Cebpb*.

### 2.4. Inhibition of Osteoblast Wnt Signaling Inhibits ST2 Cells Differentiation

**Figure 4:**
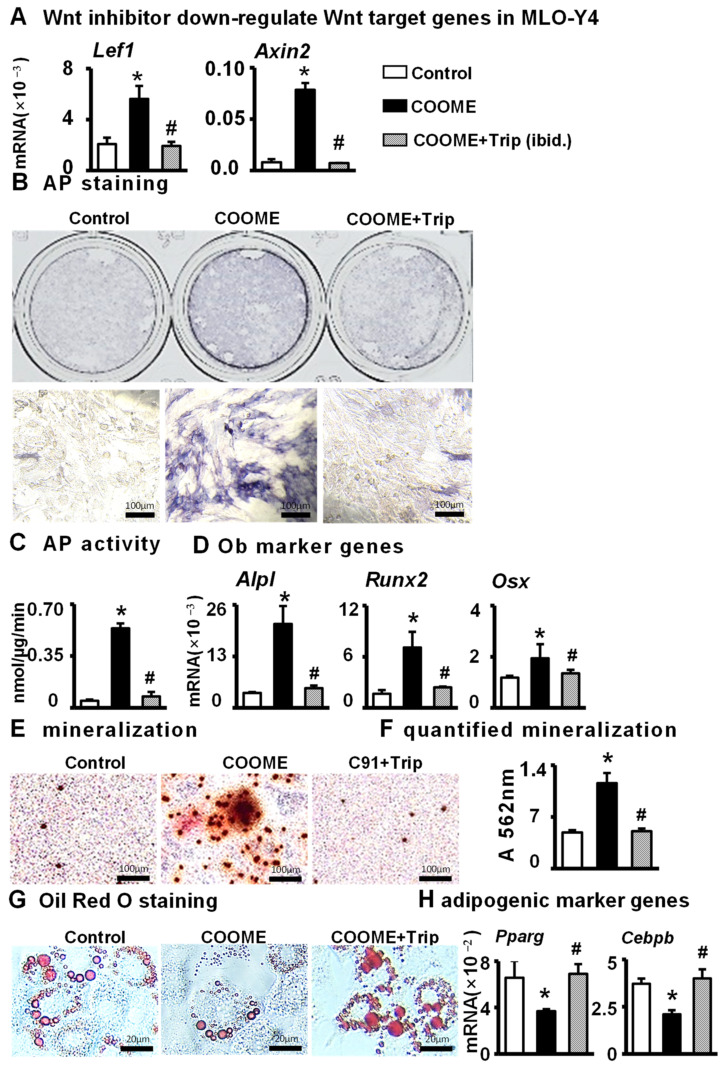
Effects of COOME treated by triptonide on osteoblast differentiation. Tript is a Wnt signaling inhibitor. COOME was treated with or without Wnt inhibitor (50 nM) for 24 h. (**A**) qPCR to detect the expression of Wnt target genes in MLO-Y4 cells after treatment. The treated COOME were then co-cultured with ST2 cells for 3 days and then detected by osteogenic differentiation assay. (**B**) AP staining analysis (**C**) AP biochemical quantitative analysis. Scale bar = 100 µm. (**D**) qPCR detection of osteogenic target genes mRNA expression levels. (**E**) Alizarin red S staining. Scale bar = 100 mm. (**F**) Mineralization quantification assay. (**G**) Oil red O staining. Scale bar = 20 µm. (**H**) qPCR detection of mRNA expression of adipogenic differentiation marker genes. Results are expressed as mean ± SD (n = three per group). * indicates *p* < 0.05, vs. the control group. # indicates *p* < 0.05, vs. the COOME group.

We next determined whether COOME plays a biological role in promoting osteoblast differentiation and inhibiting adipogenic differentiation through canonical Wnt signaling. We used triptonide to inhibit Wnt signaling in C91-activated osteocytes. The qPCR results showed that the levels of the Wnt target genes *Lef1* and *Axin2* in the inhibition group were significantly decreased (Figure 4A), almost to the levels in the control group, compared with the levels in the C91 treatment group. Subsequently, the effects of COOME on the levels of osteogenic differentiation and mineralization of ST2 cells were also eliminated by triptonide (Figure 4B–F). Furthermore, triptonide reversed the inhibitory effect of COOME on the adipogenic differentiation of ST2 cells (Figure 4G,H), indicating that COOME promoted the osteogenic differentiation and mineralization and inhibited adipogenic differentiation of ST2 cells through the canonical Wnt signaling pathway.

### 2.5. COOME Promotes Cell Proliferation and Does Not Affect Cell Survival in PCI3D Modules

**Figure 5:**
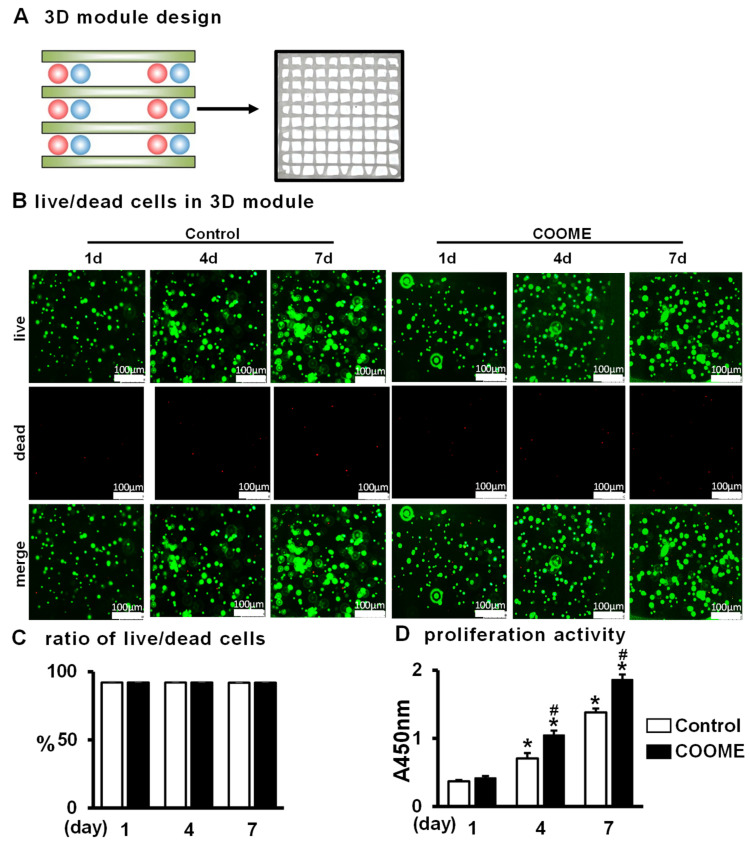
Cell survival and proliferation of ST2 cells co-cultured with COOME in 3D modules. (**A**) Schematic diagram of a PCI3D system design (**left**) and its printing module image (**right**). The COOME-loaded hydrogel bundles, the ST2-loaded hydrogel bundles, and the PCL bundles were printed with each other to form one layer, and four layers were continuously printed to form a 3D module. (**B**–**D**) The survival and proliferation of cells 1, 4, and 7 days after were detected by live/dead cell staining. The green indicates live cells and the red indicates dead cells. Scale bar = 250 µm. Results are expressed as mean ± SD (n = three per group). * indicates vs. 1 day by one-way ANOVA, # indicates vs. the control group, *p* < 0.05.

To verify whether COOME has an osteogenic function in vivo, we used the PCL and cell-integrated 3D printing system developed by our laboratory to construct the PCI3D functional module in order to mimic the real in vivo osteogenic process. At the bottom of the module, PCL bundles were printed first, and then mixed GelMA hydrogel bundles loaded with COOME and ST2 cells were printed. These sections were alternately printed for a total of four layers (Figure 5A). The PCI3D module had stable support performance and a suitable pore size of the connecting channel, which was conducive to the transport of nutrients and metabolites.

The PCI3D module loaded with COOME/ST2 was cultured for 1, 4, or 7 days. Calmodulin AM and PI staining for live/dead cells showed that the cell survival rates in the functional modules of the experimental group and the control group were as high as 89.5% (Figure 5B,C). The CCK8 proliferation activity assay showed that the proliferation ability of all cells in the PCI3D functional module was significantly increased (Figure 4D). On the fourth and seventh days, the proliferation activity of the COOME group was higher than that of the control group. These results show that the PCI3D function module can provide a good environment for cell survival and proliferation and that including the COOME group in PCI3D modules can also promote the proliferation of ST2 cells compared with that under control conditions.

### 2.6. COOME Promotes the Osteogenic Differentiation and Mineralization of ST2 Cells in PCI3D Modules

**Figure 6:**
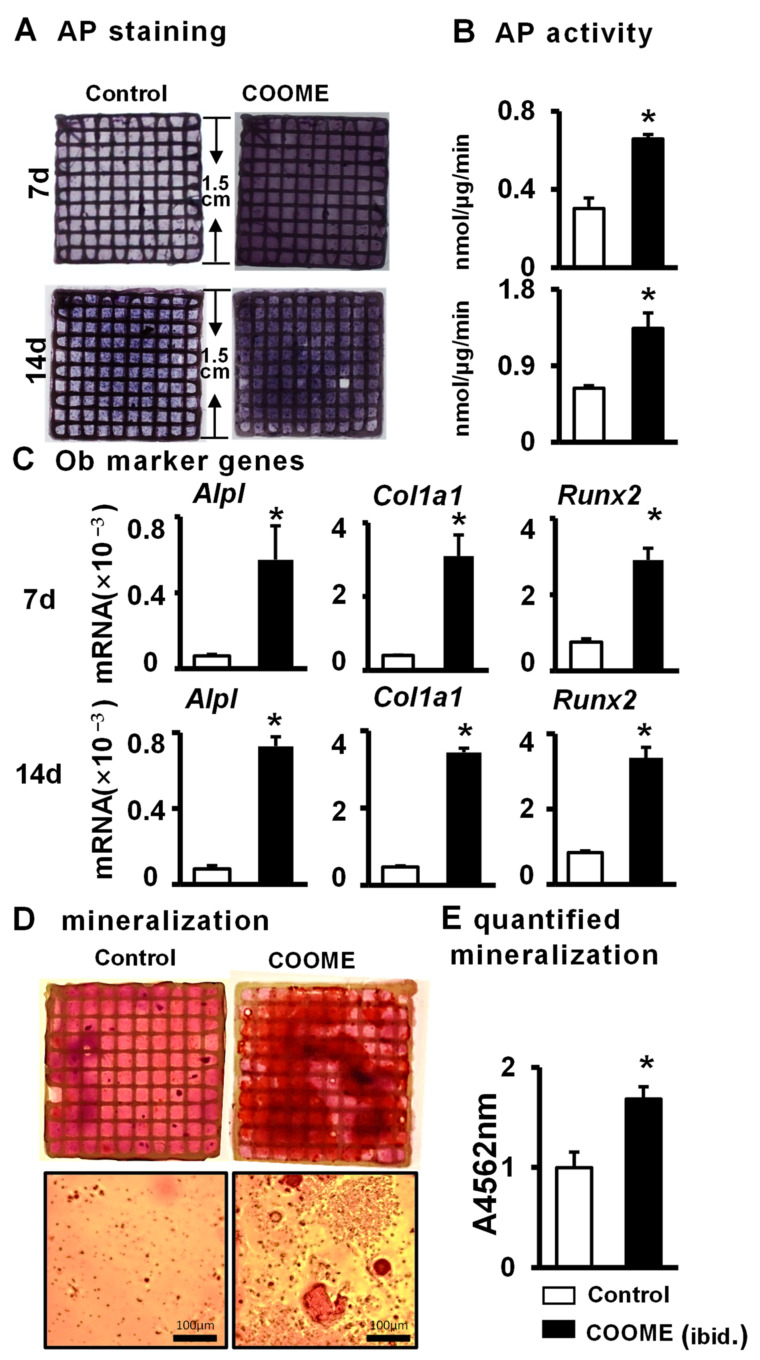
The effects of COOME on ST2 cell differentiation and mineralization in 3D modules. The 3D modules were cultured in the complete medium, changing half of the medium every two days, for 7 and 14 days. (**A**) AP staining analysis. (**B**) AP biochemical quantification analysis. (**C**) qPCR detection of the effect of COOME in the 3D module on the expression of osteogenic target genes in ST2 cells. The 3D module was cultured in the complete medium for 3 days and then replaced with the osteogenic induction medium. (**D**) Alizarin red S staining. Scale bar = 100 µm. (**E**) Mineralization quantitative analysis. Results are expressed as mean ± SD (n = 3 per group). * Indicates *p* < 0.05, vs. the control group.

We have demonstrated that the PCI3D functional module is conducive to cell survival and proliferation. Next, the PCI3D module containing COOME/ST2 was cultured to measure osteogenic differentiation after 7 days and 14 days. AP staining and biochemical quantitative results showed that COOME significantly increased the osteogenic differentiation ability of ST2 cells in the PCI3D module compared with that of the control group (Figure 6A,B). The qPCR results showed that the expression of the osteoblast marker genes *Alpl*, *Col1a1*, and *Runx2* in the COOME group was much higher than that in the control group (Figure 6C). Subsequently, we examined the effect of COOME on the formation of bone mineralized nodules in the PCI3D module. The PCI3D module was cultured in complete medium for 7 days and then replaced with osteogenic induction medium for 21 days. Alizarin red S staining and quantitative analyses showed that the COOME module formed larger and denser calcium nodules (Figure 6D,E), which substantiated the positive effect of COOME on ST2 cell mineralization.

### 2.7. COOME Promotes Angiogenesis

**Figure 7:**
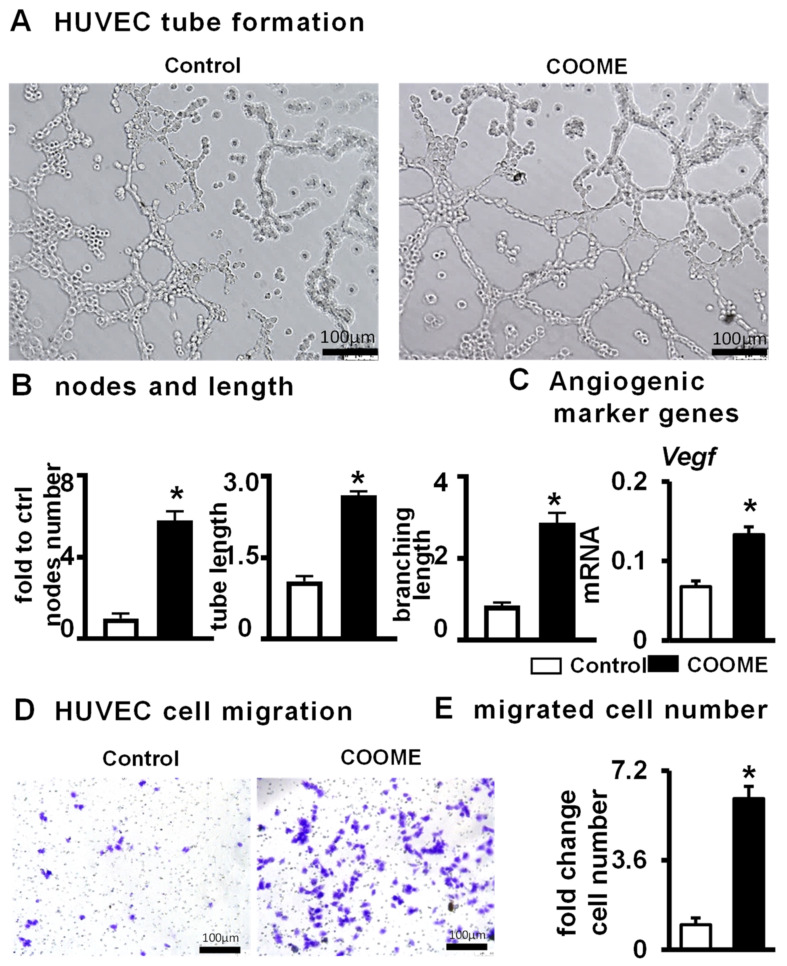
The effect of COOME on angiogenesis. (**A**) Images of tube formation in HUVECs after co-culture of COOME and ST2 cells for 6 h. (**B**) Calculation of formed nodes and tubule lengths. (**C**) Angiogenic markers. (**D**,**E**) COOME was used to image and quantify the migrated cells cultured in the transwell chamber for 48 h. Scale bar = 100 µm. Results are expressed as mean ± SD (n = three per group). * Indicates *p* < 0.05, vs. the control group.

Blood vessels play a critical role in bone homeostasis and repair [30]. To investigate whether COOME can induce angiogenesis, we measured angiogenesis after the co-culture of COOME and HUVECs. Compared with the control group, COOME increased the formation of vascular tubules (Figure 7A) and increased the number of tubular nodes, and the total length and number of branches increased by 5.8 times, 2.7 times, and 2.9 times, respectively (Figure 7B). We also found that the gene expression level of *Vegf* was higher after COOME treatment than after DMSO treatment (Figure 7C). In addition, compared with the control group, COOME facilitated the migration of HUVECs (Figure 7D); specifically, it increased the number of migrated cells by 6.1 times (Figure 7E). These results indicate that COOME has a certain pro-angiogenic function.

### 2.8. COOME-Conditioned Medium Promotes Osteoblast Differentiation and Angiogenesis

**Figure 8:**
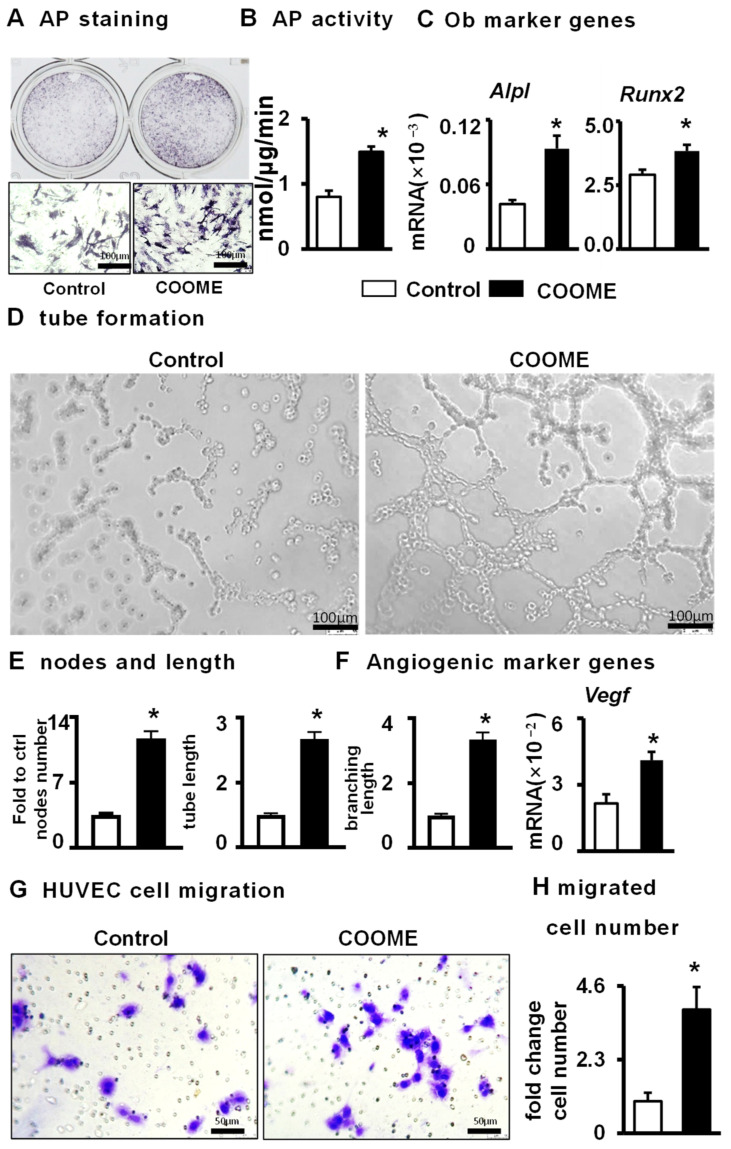
The effects of COOME-conditioned medium on osteoblast differentiation and angiogenesis. The conditioned medium was collected from the supernatant of the COOME medium and control groups were cultured for 24 h. (**A**–**C**) Effect of conditioned medium on osteoblast differentiation of ST2. (**D**–**F**) Effects of conditioned medium on angiogenesis. Scale bar = 100 µm and scale bar = 50. (**G**,**H**) Endothelial cell migration assay. Scale bar = 100 µm. Results are expressed as mean ± SD (n = three per group). * Indicates *p* < 0.05 vs. the control group. n = 3.

We demonstrated that COOME can promote the osteogenic differentiation of ST2 cells and promote angiogenesis by direct contact with cells. To study whether COOME has an indirect effect on cells, we used C91 to treat MLO-Y4 cells for 24 h, then withdrew the drug, replaced the complete medium, and cultured the cells for 1 day. The conditioned medium of COOME was collected and used to treat ST2 cells and HUVECs. AP staining, AP biochemical quantification, and osteoblast marker gene expression analysis showed that the conditioned medium promoted ST2 cell differentiation (Figure 8A–C). In addition, compared with DMSO, the conditioned medium led to the formation and an increased number of tubules. The number of tubular nodes, the total length of tubules, and the number of branches in the conditioned medium of COOME were augmented by 11.6 times, 3.3 times, and 4.2 times, respectively (Figure 8D–F). Moreover, compared with the control group, the conditioned medium group exhibited greater HUVEC migration (Figure 8G), with 3.9 times the number of migrated cells (Figure 8H).

## 3. Discussion

The construction of bone implants with self-metabolizing biological activity and mechanical support is a frontier topic in the field of orthopedics. Currently, the cells in porous scaffolds carrying living cells are only attached to the surface of the material, so the available system cannot truly simulate natural bone tissue. In our laboratory, we found that Wnt-activated osteocytes may affect bone formation by influencing surrounding skeletal cells through paracrine signaling, and specific data showed that Wnt-activated osteocytes promote bone formation, bone resorption [15], and stem cell proliferation. These findings have translational applications [21,31,32] and will be beneficial for BTE. We found that C91 effectively prevented the degradation of β-catenin in the cytoplasm of MLO-Y4 osteocytes and mediated β-catenin entry into the nucleus, thereby significantly increasing the expression of Wnt target genes. MLO-Y4 cells treated with C91 for 24 h significantly increased the osteogenic differentiation and mineralization of ST2 cells, indicating that C91-treated osteocytes can form an osteogenic microenvironment (COOME), and these effects were reversed by triptonide. Subsequently, we used PCI3D technology to reconstruct a functional module by using the new design of hard scaffold materials such as PCL integrated with cells to simulate osteogenesis in vivo and confirmed the function of COOME in the 3D bioprinting module. In this process, we demonstrated that COOME provided a good environment for ST2 cells; the cell survival rate was as high as 92%, and it significantly promoted proliferation after 7 days of in vitro culture. Unlike in previous studies, COOME not only promoted osteogenic differentiation and mineralization for up to 28 days, but also inhibited adipogenic differentiation. In addition, COOME also recruited endothelial cells and promoted angiogenesis. Our study integrates COOME with osteogenic function into our new PCI3D design, which has potential application value for BTE.

In the field of BTE, the emergence of 3D bioprinting technology has provided the possibility for the accurate construction of implants and the regeneration and repair of bone defects [5]. Because it is able to create porous structures with interconnected pores, live cell printing can successfully achieve the adaptation of individualized shapes of tissue engineering scaffolds, which creates a suitable biological microenvironment for cell adhesion, proliferation, and differentiation [13,33,34]. Although 3D printing technology has solved the problem of material shape matching, the bioactivity of BTE materials still needs to be improved. Therefore, various studies are currently aimed at creating a scaffold with mechanical support and safe biological activity that can mimic natural bone tissue [35,36]. However, the development of the bionic microenvironment is a challenge for the entire industry. In the early stages of our research, we found that the bone cell acellular matrix that was activated by Wnt signaling via gene manipulation accelerated the repair of critical-sized parietal bone defects by promoting osteoblast formation, angiogenesis, and neurogenesis [21]. Moreover, GelMA/PCL scaffolds containing ST2 cells pretreated with Wnt3a can effectively enhance the osteogenic and angiogenic activities of bone repair biomaterials in vitro and in vivo [32]. Our team has proven that this functional module has osteogenic and vascular functions, solves the problem of biological activity, and can be used as a good alternative material for bone regeneration and bone repair. However, the limitations of gene manipulation make it difficult to apply to large-scale clinical transformation. The greatest innovation of this research is that it uses a safe and effective small molecule drug, C91, rather than relying on gene manipulation. Moreover, we found that COOME is a safe and effective osteogenic microenvironment that not only induces target cell differentiation in vitro to facilitate bone angiogenesis, but also has a survival rate of up to 92% after integration with PCI3D. COOME mimics in vivo environments to a great extent and can hopefully be used to build functional repair modules for further clinical transformation.

It has become necessary to vigorously carry out basic research on the bone formation microenvironment, as well as to develop bioactive materials with biomimetic functions, induce stem cell differentiation, and complete bone regeneration and repair. The cells involved in bone regeneration are mainly osteoblasts, osteoclasts, and osteocytes, the latter of which can regulate osteoclasts and osteoblasts to control bone reconstruction [37]. Osteocytes, as the main cells controlling the bone microenvironment, are the target cells that mediate Wnt/β-catenin signaling to induce bone anabolism [15,38]. Osteocytes account for the vast majority of skeletal cells (>90%), and their strategic position is very important. These cells can keenly perceive local/systemic mechanical and signal stimuli and induce a systemic response. Previous studies have shown that direct oral or in vivo administration of Wnt agonists can increase bone mass, but these administration methods may activate the systemic Wnt signaling and easily cause unknown systemic side effects [39,40,41]. In this study, we focused on osteocytes and treated them with C91 for 24 h to activate Wnt signaling. After removing these drugs, osteocytes still had vigorous biological activity; for example, they were able to promote the osteogenesis and angiogenesis of ST2 cells. In contrast, Bertacchini et al. [42] reported that the conditioned medium of the C91-pretreated osteocyte cell line MLO-Y4 inhibited the differentiation ability of 2T3 cells. Unlike us, they used a drug concentration of 100 nM, while the optimal concentration we used was 5 μM; in addition, their cells were cultured for 5 days, while our osteogenic differentiation time was 3 days and the mineralization time was 14 days. Furthermore, they did not mention whether C91 was withdrawn after the treatment of osteocytes, nor did they mention the method of collecting the conditioned medium. One of the highlights of this study is that COOME is drug-free, meaning that it does not affect other cells or tissues around it; COOME acts only on osteocytes and can be safely used for BTE. At present, research on the function and mechanism of osteocytes is emerging, which will be conducive to uncovering the medical function of Wnt signaling in osteocytes.

In our study, we found that COOME can promote ST2 cell proliferation, osteogenic differentiation, and mineralization in PCI3D modules. Generally, the cells started to differentiate after exiting the cell cycle. The exception is stem cells, which have two main properties: they both self-renew and differentiate when they divide [43]. Many studies have reported that MSCs can promote both proliferation and differentiation. However, He et al. reported that Emdogain promotes osteoblast (MC3T3-E1) proliferation and differentiation [44]. Among them, Prasadam et al. demonstrated that osteocytes can promote HUVEC cell proliferation and differentiation [45], showing that non-stem cells can also proliferate and differentiate under certain stimuli. In recent years, there have been more reports of simultaneous cell proliferation and differentiation in bone tissue engineering [46,47], for example, Guo et al. reported that ND/PCL fibrous matrices can promote the proliferation and differentiation of MC3T3-E1 cells. Zamani et al. showed that chemically surface-modified and RGD-immobilized 3D-printed poly(ε-caprolactone) scaffolds promote the proliferation and osteoblasts of MC3T3-E1 pre-osteoblasts [48]. These studies believe that the selection and optimization strategy of bioprinting materials is the key to improving cell biocompatibility (including cell survival and proliferation) and biological function.

Recent research has shown that bone formation and fat formation are antagonistic to each other [49,50]. However, thus far, these studies have only focused on stem cells and osteoblasts, and the role of osteocytes in adipogenic differentiation has not been reported. We found that after COOME treatment of ST2 cells, the amounts of lipid droplets decreased, and the mRNA expression levels of the adipogenic marker genes *Pparg* and *Cebpb* decreased, indicating that COOME inhibited adipogenesis. This result is consistent with the previous results in stem cells: the Wnt/β-catenin pathway induces osteogenesis and inhibits adipogenesis [51], whereas *Pparg* enhances adipogenesis while inhibiting osteogenesis [52]. In addition, Wnt10b inhibits *Cebpb* and *Pparg* in ST2 cells, leading to increases in the levels of osteogenic transcription factors [51]. We used the Wnt inhibitor triptonide to reverse the inhibitory effect of COOME on adipogenic differentiation and verified our results. We speculate that C91 can inhibit adipogenic differentiation, promote osteogenesis, and reduce *Pparg* and *Cebpb* expression in ST2 cells by activating Wnt signaling in osteocytes.

Angiogenesis is closely related to the success of bone regeneration [53], and blood vessels play an indispensable role in bone formation. Blood vessels are not only transport organs for nutrient diffusion, cell proliferation, and new bone tissue growth, but also play key roles in regulating cells and signaling molecules involved in bone regeneration [8,54]. *Vegf* can effectively promote the coupling of angiogenesis and bone formation in bone repair [55]. Previous studies have reported that classical and nonclassical Wnt signaling pathways promote angiogenesis by affecting the proliferation and migration of endothelial cells [56]. Dharmarajan et al. [55] found that activation of the Wnt signaling pathway can lead to an upregulation of *Vegf* expression and induce angiogenesis, while inhibition of the *Vegf* signaling pathway inhibits angiogenesis. Our results showed that COOME can promote the formation of vascular endothelial tubules in vitro, which may be achieved by upregulating *Vegf*; meanwhile, the Wnt/β-catenin signaling inhibitor triptonide can reduce tubule formation in vitro. This discovery further proves that the functional modules containing COOME can promote endothelial cell differentiation and bone formation. In the next study, we will implant the COOME/ST2/PCL scaffold into mice and rats for bone defect repair.

Overall, our self-developed hard material with COOME/ST2 integrated 3D printing will provide new ideas for the development of bone repair materials and promote clinical translation and application.

## 4. Materials and Methods

### 4.1. Reagents and Cells

#### 4.1.1. Chemicals

Wnt signaling agonist CHIR99021 (C91) and Wnt signaling activator triptonide (Tript) were obtained from MedChemExpress (Shanghai, China), and Lyophilized gelatin methacryloyl (GelMA) and Lithium phenyl (2,6-trimethyl benzoylzoyl) phosphinate (LAP) were obtained from SunP Biotech (Beijing, China). Polycaprolactone (PCL, average MW 45000) and RIPA lysis buffer were obtained from Sigma-Aldrichh (St Louis, MO, USA), Trizol from AccurateBiology (Hunan, China), and Indomethacin and Hydrocortisone from Solarbio Biotechnology (Beijing, China). Matrigel was from Corning (Corning, NY, USA). Alizarin red S staining kit and Oil red O saturated solution were obtained from Solarbao Biotechnology (Beijing, China), and 3-Isobutyl-1-methylxanthine (IBMX) was obtained from Calbiochem (Shanghai, China).

#### 4.1.2. Assay Kits

BCIP/NBT alkaline phosphatase (AP) color-rendering kit, alkaline phosphatase activity assay kit, bicinchoninic acid (BCA) protein assay kit live/dead viability assay kit, crystal violet staining solution, and DAPI staining solution were obtained from Beyotime Biotechnology (Shanghai, China). AG RNAex Pro reagent and reverse transcription reaction kits were obtained from AccurateBiology (Changsha, Hunan, China), and cell counting kit-8 was from MedChemExpress (Shanghai, China).

#### 4.1.3. Antibodies

Polyclonal anti-β-catenin antibody was supplied by Wanlei (Shenyang, Liaoning, China), along with FITC labeled goat anti-rabbit IgG (H + L) (Shanghai, China).

#### 4.1.4. Cell Culture Reagents and Cell Sources

Fetal bovine serum (FBS) and α-modified MEM (α-MEM) were purchased from Gibco (Maryland, Gaithersburg, USA), while pancreatin and penicillin-streptomycin antibiotics (PS) were from Beyotime (Shanghai, China). Human umbilical vein endothelial cells (HUVECs) was obtained from ATCC (Manassas, VA, USA). MLO-Y4 was provided by Lynda Bonewald. ST2 cells were provided by Dr. Steve Teitelbaum.

### 4.2. Cell Culture

The culture of murine primary cell line ST2 and osteocyte cell line MLO-Y4 was performed as previously reported [31]. The cells were cultured with α-MEM medium (Gibco, Billings, MT, USA) containing 10% fetal bovine serum (FBS, BI, Israel) with 50 U/mL PS at 37 °C and 5% CO_2_. Distinctively, HUVECs were cultured with Dulbecco’s modified Eagle’s medium (DMEM). MLO-Y4 and ST2 or HUVEC cells were plated in 24-well plates at 2 × 10^4^ and 8 × 10^4^ cell densities and co-cultured for 3 days, respectively.

### 4.3. Activation and Inhibition of Wnt Signaling in Osteocytic MLO-Y4

C91 works as a Wnt activator [57] to activate the Wnt signal pathway of MLO-Y4 for 24 h. Meanwhile, the control group is the MLO-Y4 treated by DMSO. Triptonide was used to inhibit the Wnt signal pathway by binding to the C-terminal transactivation domain of β-catenin to silence β-catenin action [58]. An amount of 50 nM of triptonide and 5 µM of C91 were added at the same time to act on the MLO-Y4 to block the activation of Wnt signaling.

### 4.4. PCL and Cell-Integrated 3D Printing

GelMA was fully dissolved in α-MEM, and 20% (*w*/*v*) of GelMA solution containing 0.5% (*w*/*v*) LAP was prepared and stored at 4 °C for use. A total of 2 × 10^5^ MLO-Y4 cells and 8 × 10^5^ ST2 cells were mixed and suspended in 0.5 mL of α-MEM. GelMA was mixed with the prepared cell suspension and placed in a syringe to cool at 4 °C for later use, ready for printing.

Then, the polycaprolactone particles were loaded into the preheated hard material nozzle, and at 95 °C, the molten PCL was printed into a PCL beam at a speed of 2 mm/s to form a scaffold frame with a PCL beam diameter of 400 μm and an interbeat beam interval of 1100 μm. Immediately after 25 °C, the suspension of GelMA cell mixing was printed on the scaffold frame at a printing speed of 5 mm/s and parallel PCL beams were placed into a 300 μm diameter cell bundle with a 500 μm spacing between the bundles. After the complete printing of a layer of PCL beam and cell beam, GelMA hydrogel was irradiated with 405 nm blue light for 10 s to crosslink and solidify, and then the second layer was repeatedly printed in the direction of the vertical first PCL/cell bundle to form a 0/90° support structure. A total of four layers needed to be printed, and finally, a PCI3D module was obtained. The module was then placed in a 6-well plate and incubated in 5% CO_2_ at 37 °C for use.

### 4.5. Ex Vivo Assay for Osteoblast Differentiation and Matrix Mineralization on Two- or Three-Dimensional Levels

To test the function of MLO-Y4 treated with C91 on ST2 osteogenic differentiation, we co-cultured MLO-Y4 with ST2, which was tested by AP staining, biochemical activity assay, and matrix mineralization assay, as described [31]. The osteoblast differentiation function of MLO-Y4 treated with C91 on ST2 in the 3D module was tested by the above methods.

#### 4.5.1. Alkaline Phosphatase Staining

Alkaline phosphatase staining (AP staining) was performed as previously described [31,32]. After 3 days of cell culture and aspirating the medium, the cells were washed with PBS (Sorlabio, Beijing, China) two times to remove any interference with proteins contained in the serum. Then, the 3.7% formaldehyde (Chuandong, Chongqing, China) was used to fix the cells for 5 min at room temperature. AP staining solution was preconfigured and then added into platelets for 30 min according to the instruction of the BCIP/NBT alkaline phosphatase color development kit. Alternatively, the AP staining cells in the module could be performed after 7 and 14 days of cell culture, and the staining time could be extended to four hours. The staining results were reported by a digital camera.

#### 4.5.2. AP Biochemical Activity Assay

The AP biochemical activity assay was executed as previously reported [31,32]. After aspirating the medium and washing the cells with PBS, 300 µL 10 mM Tris/HCl (pH 7.4) was added to each well. Then, cells were starched from the plate by the tip of the gun and blown repeatedly. The cell suspension was transferred to an EP tube for ultrasound. Then, the supernatant was retained and centrifuged at 12,000 rpm for 3 min to remove the cell debris. Finally, the supernatant was used for assay with the AP detection kit (Beyotime, China).

#### 4.5.3. Mineralization Assay (Alizarin Red S Staining)

The 3D modules were cultured in a growth medium for a week, and then bone nodule formation was induced in osteogenic medium containing 0.1 mM dexamethasone, 10 mM β-glycerophosphate disodium salt solution, and 50 μg/mL L-ascorbic acid for 14 days. Matrix mineralization was analyzed by alizarin red S staining as reported [21].

### 4.6. Cell Proliferative Activity

To evaluate the effect of C91 on osteocyte MLO-Y4 proliferation, the cells were seeded in 96-well plates (5000 per well) and adhered overnight. The proliferation of osteocyte MLO-Y4 treated with different concentrations of C91 (0, 10, 100, 500 nM; 1, 2.5, 5, 10 µM) was tested by cell counting kit-8 according to the previous method [25,59,60]. After aspirating the medium, cells were washed with PBS two or three times in a 96-well plate. An amount of 100 µL complete medium containing10 µL CCK-8 was added to each well, and then the plate was incubated for 1–3 h. Subsequently, the absorbance at 450 nm of the supernatant in the incubated plate is measured with a microplate reader. It is worth noting that in order to evaluate the proliferation activity in the PCI3D modules on days 1, 4, and 7 after initiation of culture, the PCI3D modules were cut into four pieces and placed in a 96-well plate. The next steps are the same as before.

### 4.7. Cell Viability Assay

The live/dead viability assay kit was used to detect the cell viability in PCI3D modules as previously reported [21]. After 1, 4, and 7 days of cell culture in PCI3D modules, the medium was aspirated and PBS was used to wash cells. Then, these cells were incubated in the staining mixture, which contained 1 µL Calcein AM (1000×), 1 µL PI (1000×), and 1 mL assay buffer. The plates were put in the incubator for 30 min and the results were reported by using an inverted fluorescence microscope (Leica, Wetzlar, Germany). Finally, the ImageJ (64-bit, v1.46) software was used to detect cell viability.

### 4.8. RNA Extraction and Gene Expression Analysis

The total RNA of cells was extracted using the TRIzol reagent, and cDNA was synthesized from the total RNA extraction through a reverse transcription reaction kit (TaKaRa, Shiga, Japan) [15]. The synthesized cDNA was 5-fold diluted to be stored until used as templates for qPCR to detect genes with a primer set (Table 1). The relative expression levels of mRNAs were normalized to the housekeeping gene GAPDH by using the 2-ΔCT method.

### 4.9. Conditioned Medium

The conditioned medium was prepared as previously reported [31]. Briefly, MLO-Y4 cells were treated with C91 for 24 h, then the cells were washed in PBS and continuously cultured in a C91-free medium for another 24 h. At the end of the treatment, the supernatant was collected and stored at −80 °C until use. In parallel, the control supernatant was collected from MLO-Y4 treated with DMSO. The conditioned medium was prepared by mixing 800 µL of 10% FBS αMEM with 200 µL conditional medium per 1 mL.

### 4.10. HUVEC Tube Formation Assay In Vitro and Transwell Migration Assay

A concentration of 1.2 × 10^5^ cells/cm^2^ HUVECs and 0.3 × 10^5^ cells/cm^2^ MLO-Y4 cells treated with DMSO or C91(or 1.5 cells/cm^2^ HUVECs cultured in a conditioned medium) were mixed and inoculated onto Matrigel (200 µL) in precoated 24-well plates and cultured for 6 h in an incubator as reported [31]. Capillary-like structures were captured by a phase contrast microscope. The network structures formed from HUVEC cells were quantified by ImageJ after the software automatically analyzed it, and a color-coded image appeared, in which green represented branches, orange represented master segments, blue sky represented meshes, and red was surrounded by nodes [31]. Transwell migration assay was performed as previously [31,61]. The migrated cells were imaged using a microscope and were quantified with ImageJ.

### 4.11. The Experiment of Translocation of β-Catenin into the Nucleus

MLO-Y4 cells were cultured on 24-well plates treated with C91 or DMSO. After 24 h, the nuclear localization of β-catenin in cells was detected by immunofluorescence with rabbit polyclonal anti-mouse β-catenin antibody and FITC-labeled goat anti-rabbit IgG (H + L), as described [31].

### 4.12. Adipogenic Differentiation Induction

As previously described [62], treated MLO-Y4 are co-cultured with ST2 cells in DMEM containing 0.5 μM hydrocortisone, 0.5 mM IBMX, and 60 μM indomethacin. The culture was changed every 2 days. After 10 days of culture, cells were fixed with 4% formalin, washed three times in double distilled water, and stained with oil red O solution for 10 min to detect lipid droplets by microscope (Nikon, Tokyo, Japan).

### 4.13. Statistical Analysis

Statistical analysis was performed by GraphPad Prism 8.0.1 software. The data were presented as mean ± SD. The difference between multiple groups and two independent variables was analyzed by using one-way ANOVA. *p* value < 0.05 was considered significant for all statistical tests.

## 5. Conclusions

The osteocyte cell line MLO-Y4 with Wnt signaling activated by CHIR99021 formed an osteogenic microenvironment (COOME). COOME increased the biological activity of ST2 cells in terms of cell survival, proliferation, osteoblast differentiation, mineralization, and angiogenesis, and inhibited adipogenic differentiation. This was also verified in the PCI3D module that simulated in vivo experiments, which provides valuable data for the development of BTE.

## Figures and Tables

**Table 1:** Sequences of primers used for RT-PCR (mouse).

Primer	Forward	Reverse
*Gapdh*	GCACAGTCAAGGCCGAGAAT	GCCTTCTCCATGGTGGTGAA
*beta-actin*	AGAGGGAAATCGTGCGTGAC	CCATACCCAAGAAGGAAGGCT
*Lef1*	TACCCCAGCCAGTGTCAACA	TCCATGATAGGCTTTGATGACTTTC
*Axin2*	TGCAGGAGGCGGTACAGTTC	GCTGGAAGTGGTAAAGCAGCTT
*Bmp4*	GAGGAGTTTCCATCACGAAGA	GCTCTGCCGAGGAGATCA
*Smad6*	AAGATGCTGAAGCCGTTGGT	CGAACTCCAGTATCTCCGCTTT
*Alpl*	CACGGCGTCCATGAGCAGAAC	CAGGCACAGTGGTCAAGGTTGG
*Runx2*	CCGGTCTCCTTCCAGGAT	GGGAACTGCTGTGGCTTC
*Osx*	CCCTTCTCAAGCACCAATGG	AAGGGTGGGTAGTCA TTTGCA TA
*Bglap*	CAGCGGCCCTGAGTCTGA	GCCGGAGTCTGTTCACTACCTTA
*Col1a1*	GACAGGCGAACAAGGTGACAGAG	CAGGAGAACCAGGAGAACCAGGAG
*Ibsp*	CAGAGGAGGCAAGCGTCACT	GCTGTCTGGGTGCCAACACT
*Vegf*	AGAAGGAGGAGGGCAGAATCATCAC	GGGCACACAGGATGGCTTGAAG
*Pparg*	GGAAAGACAACGGACAAATCAC	TACGGATCGAAACTGGCAC
*Cebpb*	TGAACAAGAACAGCAACGAG	TCACTGGTCACCTCCAGCAC

## Data Availability

All data generated or analyzed during this study are included in this published article.
